# Are Interstitial Cells of Cajal Involved in Mechanical Stress-Induced Gene Expression and Impairment of Smooth Muscle Contractility in Bowel Obstruction?

**DOI:** 10.1371/journal.pone.0076222

**Published:** 2013-09-30

**Authors:** Chester C. Wu, You-Min Lin, Jerry Gao, John H. Winston, Leo K. Cheng, Xuan-Zheng Shi

**Affiliations:** 1 Division of Gastroenterology and Hepatology, Department of Internal Medicine, The University of Texas Medical Branch, Galveston, Texas, United States of America; 2 Auckland Bioengineering Institute, the University of Auckland, Auckland, New Zealand; 3 Depertment of Surgery, Vanderbilt University, Nashville, Tennessee, United States of America; Temple University School of Medicine, United States of America

## Abstract

**Background and Aims:**

The network of interstitial cells of Cajal (ICC) is altered in obstructive bowel disorders (OBD). However, whether alteration in ICC network is a cause or consequence of OBD remains unknown. This study tested the hypothesis that mechanical dilation in obstruction disrupts the ICC network and that ICC do not mediate mechanotranscription of COX-2 and impairment of smooth muscle contractility in obstruction.

**Methods:**

Medical-grade silicon bands were wrapped around the distal colon to induce partial obstruction in wild-type and ICC deficient (W/W^v^) mice.

**Results:**

In wild-type mice, colon obstruction led to time-dependent alterations of the ICC network in the proximal colon segment. Although unaffected on days 1 and 3, the ICC density decreased markedly and the network was disrupted on day 7 of obstruction. COX-2 expression increased, and circular muscle contractility decreased significantly in the segment proximal to obstruction. In W/W^v^ control mice, COX-2 mRNA level was 4.0 (±1.1)-fold higher (n=4) and circular muscle contractility was lower than in wild-type control mice. Obstruction further increased COX-2 mRNA level in W/W^v^ mice to 7.2 (±1.0)-fold vs. W/W^v^ controls [28.8 (±4.1)-fold vs. wild-type controls] on day 3. Obstruction further suppressed smooth muscle contractility in W/W^v^ mice. However, daily administration of COX-2 inhibitor NS-398 significantly improved muscle contractility in both W/W^v^ sham and obstruction mice.

**Conclusions:**

Lumen dilation disrupts the ICC network. ICC deficiency has limited effect on stretch-induced expression of COX-2 and suppression of smooth muscle contractility in obstruction. Rather, stretch-induced COX-2 plays a critical role in motility dysfunction in partial colon obstruction.

## Introduction

Bowel obstruction, acute or chronic, is a significant health challenge. It may be caused by a physical blockade of the lumen (mechanical obstruction), or present as lumen dilation without an organic obstruction (functional obstruction) [1-3]. Functional bowel obstruction occurs in several gastrointestinal conditions, including achalasia, hypertrophied pyloric stenosis (gastric outlet obstruction), intestinal pseudo-obstruction, Hirschsprung’s disease, and idiopathic megacolon [3,4]. Motility dysfunction is a major pathological feature in obstructive bowel disorders (OBD) [2-4], and is responsible for symptoms such as bloating, vomiting, and constipation [2-4]. We found in a rat model of partial colon obstruction that lumen distention proximal to obstruction markedly induces expression of mechanosensitive genes such as that encoding cyclooxygenase-2 (COX-2) [5-8] selectively in smooth muscle cells (SMC). Mechanical stress-induced COX-2, through derived prostaglandins (PGs) particularly PGE_2_, plays a critical role in the suppression of smooth muscle contractility in obstruction [5,6].

Interstitial cells of Cajal (ICC) are specialized cells throughout the gastrointestinal tract, and are primarily distributed in the myenteric plexus (ICC-MY), intramuscular layer (ICC-IM), deep muscularis plexus (ICC-DM), and the submucosa plexus (ICC-SM) [9,10]. In the colon, the main types of ICC are ICC-MY, ICC-IM, and ICC-AM [9]. ICC-MY and ICC-SM have been proposed as the pacemakers of slow wave activity in gut smooth muscle, and ICC-IM and ICC-DM to mediate neurotransmission to SMC [9,10]. ICC have been also postulated as mechanoreceptors in the gut [11]. However, the exact roles of ICC in gastrointestinal physiology and pathophysiology remain controversial [12,13]. Nevertheless, studies have found that ICC number is reduced, and ICC network is disrupted in almost all OBD such as achalasia, gastric outlet obstruction, pseudo-obstruction, megacolon and severe constipation [14-18]. Some of the investigators propose that the decrease of ICC numbers and impairment of ICC networks may be responsible for the symptoms related to motility dysfunction in the disorders [15,18]. However, it remains unknown whether the ICC alterations are the cause for motility dysfunction or a result of mechanical stress in lumen dilation, a common feature in OBD.

The present study was designed to test the hypothesis that mechanical stress disrupts the ICC network in the colon and induces mechanotranscription of COX-2 in colonic smooth muscle irrespective of the presence of ICC. Our studies found that expression of COX-2 gene was profoundly induced to similar extents in wild type and ICC deficient W/W^v^ mice. However, partial colon obstruction in wild-type mice leads to time-dependent disruption of ICC in the colon. Inhibition of COX-2 improved smooth muscle contractility in obstruction in both wild type and W/W^v^ mice.

## Materials and Methods

### Ethics statement

The Institutional Animal Care and Use Committee at the University of Texas Medical Branch approved all procedures performed on the animals.

### Animals and induction of partial colon obstruction

Five- to seven-week old male W/W^v^ mice (ICC deficient WBB6F1/J-Kit ^W^/Kit ^W-v^/J, Stock number 100410) and littermate wild-type controls (WBB6F1/J(+/+)) weighing 20-28 g were obtained from the Jackson Laboratory (Bar Harbor, Maine). Mice were housed under conditions of constant temperature (23 ± 2°C) and humidity (55 ± 10%) with standard rodent chow and water ad libitum and a 12-h light/dark cycle.

Partial colon obstruction in mice was surgically induced as previously described [5,8]. Mice were anesthetized with 2% isoflurane inhalation. A midline laparotomy incision (~1.5 cm in length) was made and the distal colon was exposed. A 2 mm wide by 10 mm long medical-grade silicon band was implanted around the distal colon, approximately 2 cm from the anus [5], in wild-type and ICC deficient (W/W^v^) mice. Sham mice were also anesthetized and received surgery but the silicon band was removed immediately after being implanted. Following each procedure, antibiotics ampicillin (75 mg/kg I.M.) and analgesic buprenex (0.05 mg/kg SC) were administered. Obstruction was maintained for 1, 3 or 7 days.

### Administration of COX-2 inhibitor NS-398 in vivo

In some experiments, mice were administered daily with vehicle (200 μL of 20% DMSO) or COX-2 inhibitor, NS398 (10 mg/kg in 200 μL of 20% DMSO). In these experiments, mice received vehicle or NS398 (I.P.) immediately before the surgical operation. After the initial injection, vehicle or NS398 was administered every 24 hours until the day of euthanization. Mice were weighed daily and the amount of NS398 administered was adjusted accordingly.

### Tissue preparation

Mice were euthanized with CO_2_ inhalation, and the colon was isolated and cleansed in Kreb’s buffer (2.5 mM CaCl_2_.2H_2_O, 5.5 mM KCl, 1.2 mM MgCl_2_.6H_2_O, 15.4 mM NaHCO_3_, 1.5 mM NaH_2_PO_4_, 120.3 mM NaCl, 11.5 mM glucose). A 2 cm long segment of colon 2 cm proximal to the obstruction band was isolated, cut open, and pinned down in Kreb’s buffer in a petri dish with Sylgard base. In some animals, the distal segment to the obstruction band was also taken as self controls. The colonic muscularis externae layer was separated from the mucosa and submucosa layer, and taken for histological study, molecular measurements, and contractility determination.

### Immunofluorescence staining and quantitative analysis of c-Kit positive ICC

For tissue histology, a sheet of 1cm x 1cm colonic muscularis externae tissue was pinned flat and fixed with 100% acetone at room temperature for 30 min. After washing twice with water, the tissue was gently shaken in 1x PBS overnight at 4°C. The tissue was incubated with 0.3% Triton X-100 in PBS with 1% BSA for two hours at room temperature with gentle shaking and then washed twice with PBS plus 1% BSA for 20 minutes. To visualize the ICC, a tyrosine kinase membrane receptor characteristic of the ICC, CD117 (c-Kit), was localized using ACK2 monoclonal antibody from eBioscience (San Diego, CA). Tissue was incubated for 48-72 hours at 4°C with gentle shaking in ACK2 antibody (1:20 in PBS, 1% BSA). The tissue specimen was washed six times for seven minutes each in PBS plus 1% BSA at room temperature on a shaker. Alexa fluor 568 goat anti-rat IgG H+L (2 mg/mL Invitrogen, Carlsbad, CA) was used as the secondary antibody (1:250 in PBS, 1% BSA). After incubation for two hours at room temperature, the tissue specimens were washed five times and mounted on slides with Fluorsave for fluorescence imaging by an Olympus BX51 microscope powered by DP Controller imaging system. To ensure consistency, all slides in this project were prepared by a single lab member and all images were captured and viewed by another member. Structural properties of the ICC networks were quantitatively analyzed with the sources of the images blind to the data analyzer by an independent group at a different institute using numerical metrics for quantifying ICC network structure as described previously [19]. Unbiased thresholding algorithms were used to segment the images and determine the volume of the Kit-positive structures, thereby minimizing any human influence in the process. From the segmented images, we calculated the following characteristics of the ICC networks: density (the proportion of ICC present), thickness (the width of the cell bodies and processes), hole size (the radius of non-ICC spaces within the ICC network), and connectivity (the connectivity of the ICC network).

### RNA extraction and real-time quantitative PCR

RNA samples were extracted from colonic muscular externa tissue samples using the Qiagen RNeasy kit (Qiagen, Valencia, CA), as described previously [5-8]. SuperScript III First-Strand Synthesis System (Invitrogen, Carlsbad, CA) was used for reverse-transcription. The Applied Biosystems ABI Prism 7000 Sequence Detection System (Foster City, CA) was used for real-time PCR. The primers for mice COX-2 and 18s rRNA were obtained from Applied Biosystems. Duplicate cycle threshold (C_T_) values for the target gene COX-2 were analyzed, and 18S rRNA was used as the endogenous reference.

### Protein extraction and Western Blot

For protein extraction, the muscularis externae tissue was homogenized on ice in lysis buffer supplemented with protease inhibitors (Sigma-Aldrich, St. Louis, MO). The lysis buffer consists of (in mmol/l) 20 Tris·HCl, pH 7.5, 150 NaCl, 1 EDTA, 1 ethylene glycol-bis(β-aminoethyl ether)-N,N,N′,N′-tetraacetic acid, 2.5 sodium pyrophosphate, 1 β-glycerolphosphate, 1 Na _3_VO_4,_ and 1% Triton X-100, and 1 μg/ml leupeptin.

The proteins were resolved by standard Western blotting as described previously [5-8,20]. Equal quantities (20 μg) of total protein were loaded and run on premade 8–16% Tris-glycine SDS-PAGE (Invitrogen). They were transferred to nitrocellulose membranes (Invitrogen) for incubation with primary and secondary antibodies. Primary antibody to COX-2 (1:1,000) was obtained from Cayman Chemical (Ann Arbor, MI) [5,8]. IRDye 800-conjugated anti-mouse IgG (Rockland, Gilbertsville, PA) and Alexa Fluor 680 goat anti-rabbit IgG (Invitrogen) were used as secondary antibodies. β-Actin (1:5,000, Sigma, St. Louis, MO) was used as loading control. The detection and analysis were done by Odyssey Infrared Imaging System (LI-COR Biosciences, Lincoln, NE).

### Determination of colonic circular muscle contractility

Colonic smooth muscle strips (2 × 8 mm) were mounted along the circular muscle orientation in individual muscle baths (Radnoti Glass, Monrovia, CA, USA) filled with 10 mL carbogenated Krebs solution at 37 °C. The contractile activity was recorded as previously described [5-8,20] with Grass isometric force transducers and amplifiers connected to Biopac data-acquisition system (Biopac Systems, Goleta, CA, USA). The muscle strips were first equilibrated in the muscle bath at 1 g tension for 60 min at 37 °C. The smooth muscle contractility was tested by obtaining concentration–response curves to acetylcholine (ACh, 10^−6^–10^−2^ M) with 15 to 20 min interval between each concentration. The contractile response, expressed as area under curve (AUC) was quantified as the increase in AUCs during 4 min after addition of ACh over the baseline AUCs during 4 min before the addition of ACh. The AUC of each individual strip (g s mm^−2^) was normalized by its cross-section area (CSA). The CSA was determined using the following equation: CSA (mm^2^) = wet tissue weight (mg)/[tissue length (mm) × 1.05 (muscle density in mg mm^−3^)].

### Statistical analysis

Each data point (Mean + SEM) consists of at least three independent experiments with different animals. Student t-test was applied to compare the difference of two samples between sham and obstruction or wild-type vs. W/W^v^ mice. A p value < 0.05 was considered statistically significant.

## Results

### Obstruction leads to time-dependent alterations of c-kit positive ICC

**Figure 1:**
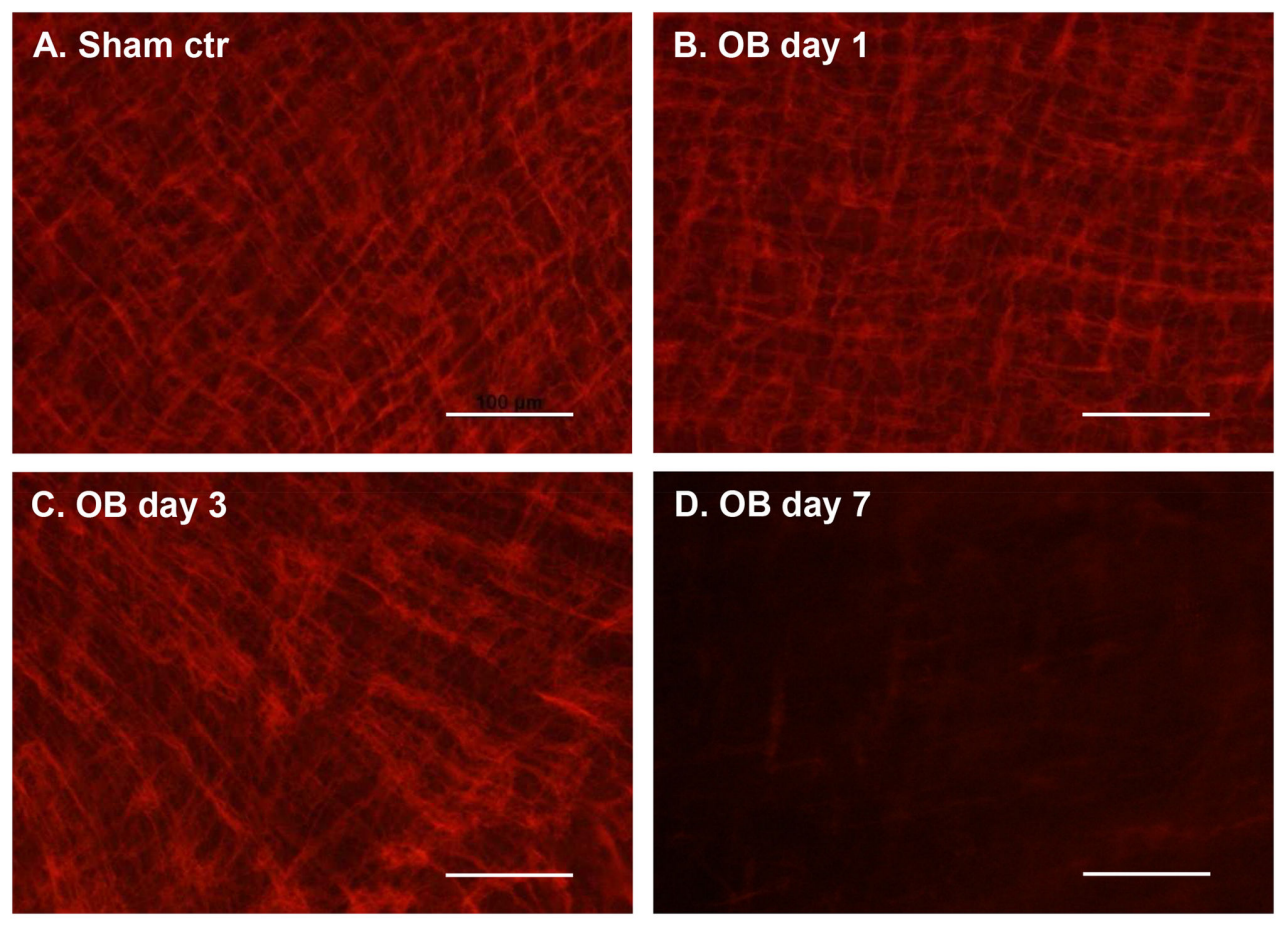
Time-dependent changes of ICC network (ICC-MY) in partial colon obstruction in mice (day 1 to day 7). ICC is labeled with immunofluorescence staining of c-Kit, a tyrosine kinase membrane receptor that is characteristic of the ICC. Representative images in A-D were captured ICC-MY staining in the colon segment proximal to obstruction band in wild-type mice with sham operation (A) and obstruction (OB) for 1 (B), 3 (C), and 7 days (D). Note that there is a significant reduction in the expression of c-Kit staining ICC by day 7 of obstruction. Images shown are representatives of three independent experiments. Calibration bars = 100 µm.

Partial bowel obstruction led to a marked dilation of the colon segment oral to obstruction. The outer circumference of the colon segment 3 cm proximal to the obstruction band increased to 18.2±0.2 mm in obstruction compared to 9.1±0.1 mm in sham on day 3 (p < 0.01, n=4). We determined the effect of lumen dilation on the ICC network (ICC-MY) in the mouse model of partial colon obstruction. Presented in Figure 1 are representative immunofluorescence images for c-Kit, a tyrosine kinase membrane receptor found in ICC, in the colon segment 2 to 4 cm proximal to the obstruction band in wild type mice. The ICC network, including cell bodies and their projections, were well organized in the ICC-MY population in the sham operated mice (Figure 1A). The integrity of the ICC network remained generally intact in obstruction at 1 day (Figure 1B), and individual cell bodies and projections were still visible. The ICC structure was minimally disrupted at 3 days of obstruction (Figure 1C). By day 7 of obstruction (Figure 1D), the ICC network was markedly altered with a drastic reduction of c-Kit positive ICC in the obstructed colon compared to that in sham control.

**Figure 2:**
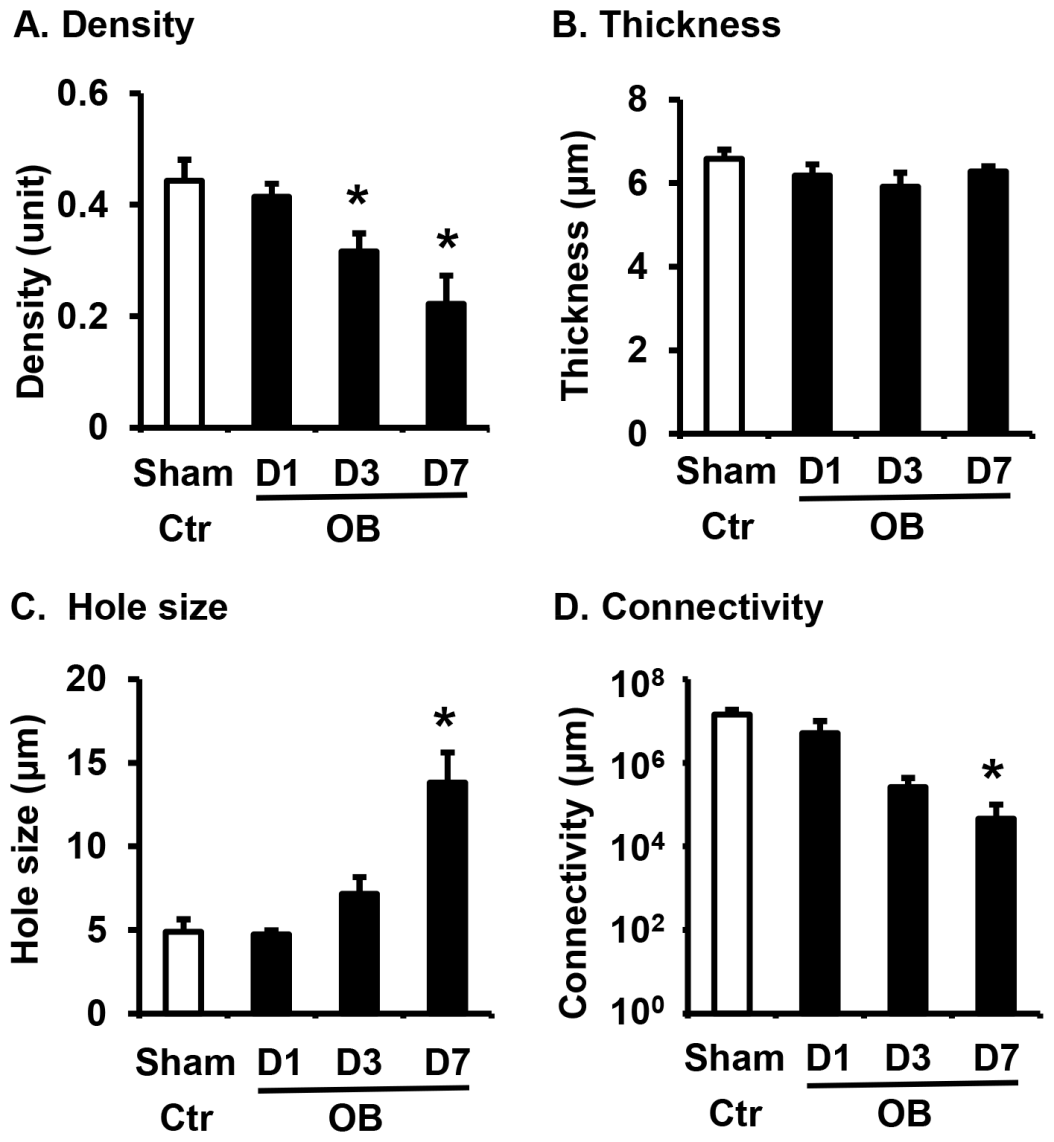
Quantitative measurements of ICC network structure in sham and obstruction (day 1 to day 7). The ICC density (A), thickness (B), hole size (C) and the ICC connectivity (D) were compared between sham control (Ctr) and obstruction (OB) on day 1, day 3, and day 7. N = 3 for each group. * p < 0.05 vs sham ctr.

Quantitative analysis of the c-kit staining [19] found that the density of c-kit positive ICC was significantly decreased in obstruction, though the thickness was not changed (Figure 2 A and B). The hole size (area without ICC structure) was significantly greater, and the overall ICC connectivity was markedly attenuated in obstruction on day 7 (Figure 2 C and Figure 2D).

**Figure 3:**
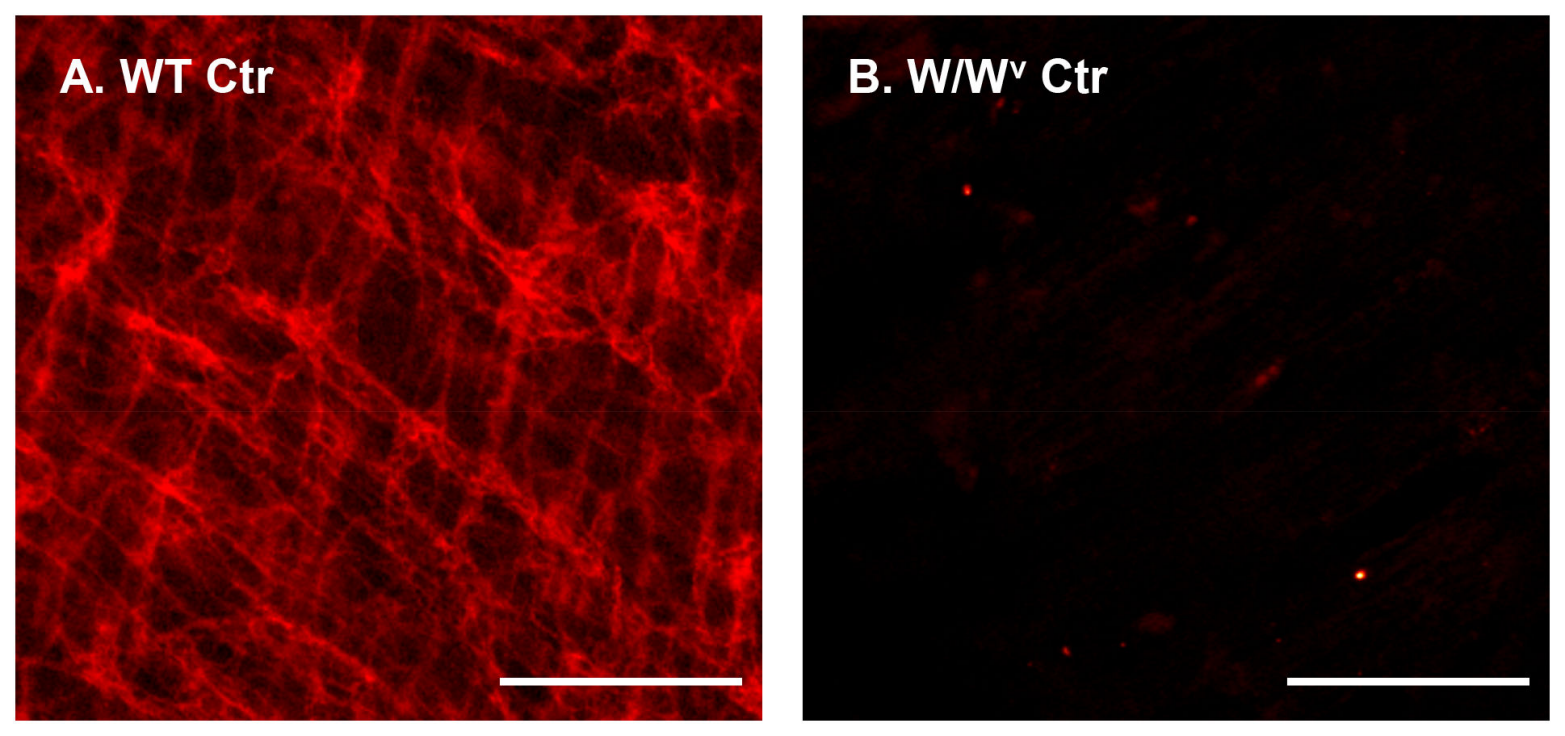
Immunofluorescence staining of ICC in W/W^v^ mice compared to wild-type mice. Note that W/W^v^ mice lack c-Kit staining ICC-MY (B) in the colon, compared to that of wild-type mice (A). Images shown are representatives of three independent experiments. Calibration bars = 100 µm.

Previous studies have demonstrated that ICC-MY disappeared in the distal colon in W/W^v^ mice [21]. Our studies with immunofluorescence staining for c-Kit also showed almost complete absence of c-kit positive ICC-MY in the distal colon of W/W^v^ mice (Figure 3). We thus did not further stain for ICC in obstruction in W/W^v^ mice.

### Obstruction induces COX-2 mRNA and protein expression in the wild-type and W/W^v^ mice

**Figure 4:**
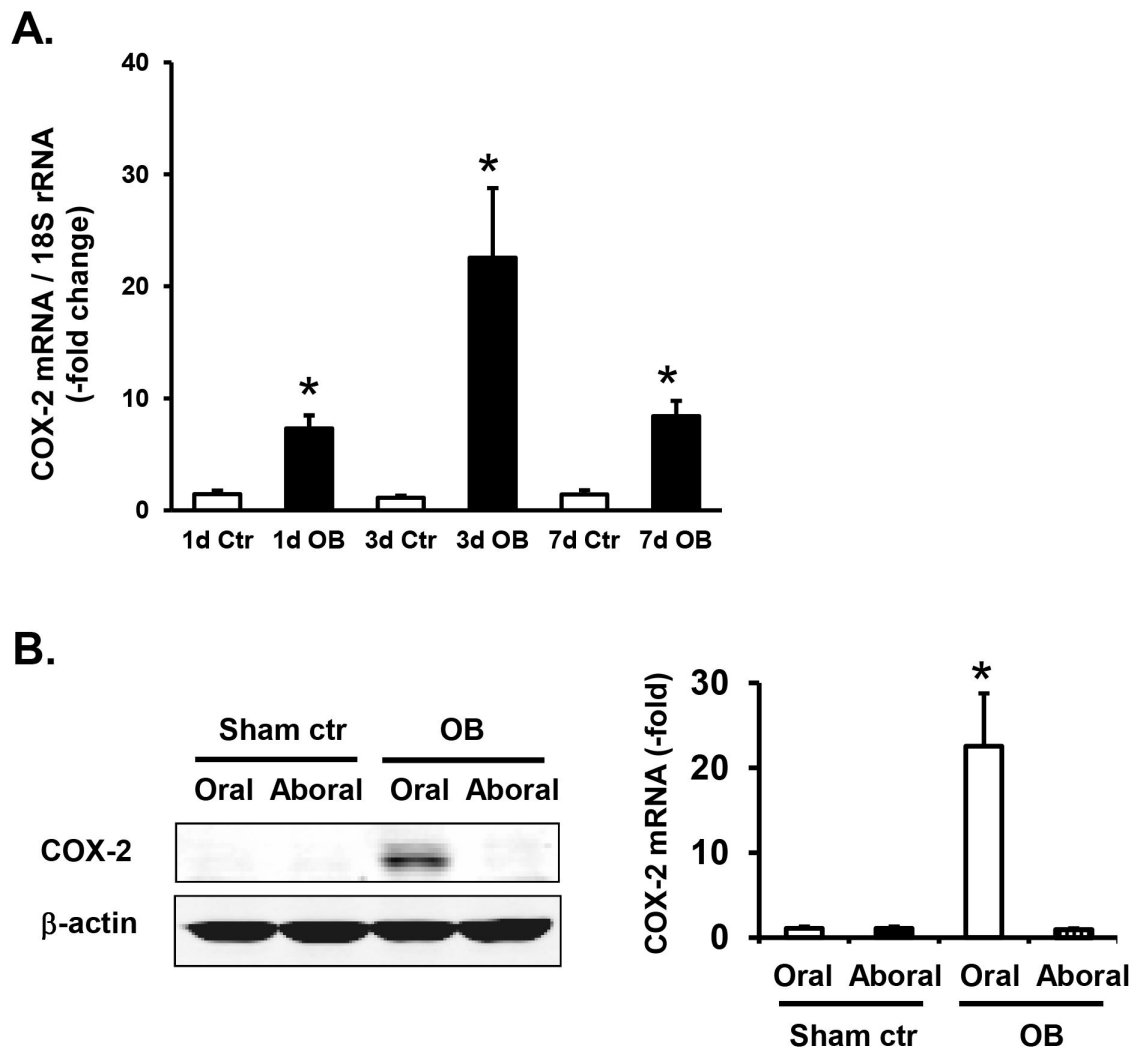
COX-2 gene expression in partial colon obstruction in wild-type mice. (A) Colonic muscularis externae was isolated from the segment proximal to obstruction in mice with sham and obstruction for different days. The COX-2 mRNA levels were measured by real-time qPCR. Note that the COX-2 mRNA expression increased in obstruction (OB) at all time points. * p < 0.05 vs. sham controls of the group. No significant difference was found among the OB groups in different days. N = 4 to 6 for each group. (B) Expression of COX-2 protein and mRNA in the segments oral and aboral to obstruction on day 3. Note that obstruction leads to a marked increase of COX-2 expression in the segment oral to obstruction, but not in the segment aboral to obstruction. * p < 0.05 vs. oral in sham controls. N = 4 to 6 for each group.

We determined the expression of COX-2 mRNA and protein in the wild-type mice in obstruction on day 1, 3, and 7. In the obstructed mice, the level of COX-2 mRNA expression was dramatically increased in the oral segment compared to that in each respective sham controls at all the time-points studied (day 1, 3, and 7) (Figure 4A). After 24 hours of colon obstruction, COX-2 gene expression increased 5.0 + 0.7-fold compared to sham (p < 0.01, N = 6 in sham and 5 in obstruction). Induction of COX-2 gene expression peaked at 3 days of obstruction, reaching a 20.3 + 5.6-fold change (P < 0.01, n = 4 for both sham and obstruction). On day 7 of obstruction, COX-2 mRNA expression was still significantly increased in tissue from obstructed mice than in sham (5.9 + 1.0-fold. p < 0.01, n = 6). Obstruction did not change the expression of COX-2 protein and mRNA in the muscularis externae of colonic segment aboral to obstruction band (Figure 4B).

**Figure 5:**
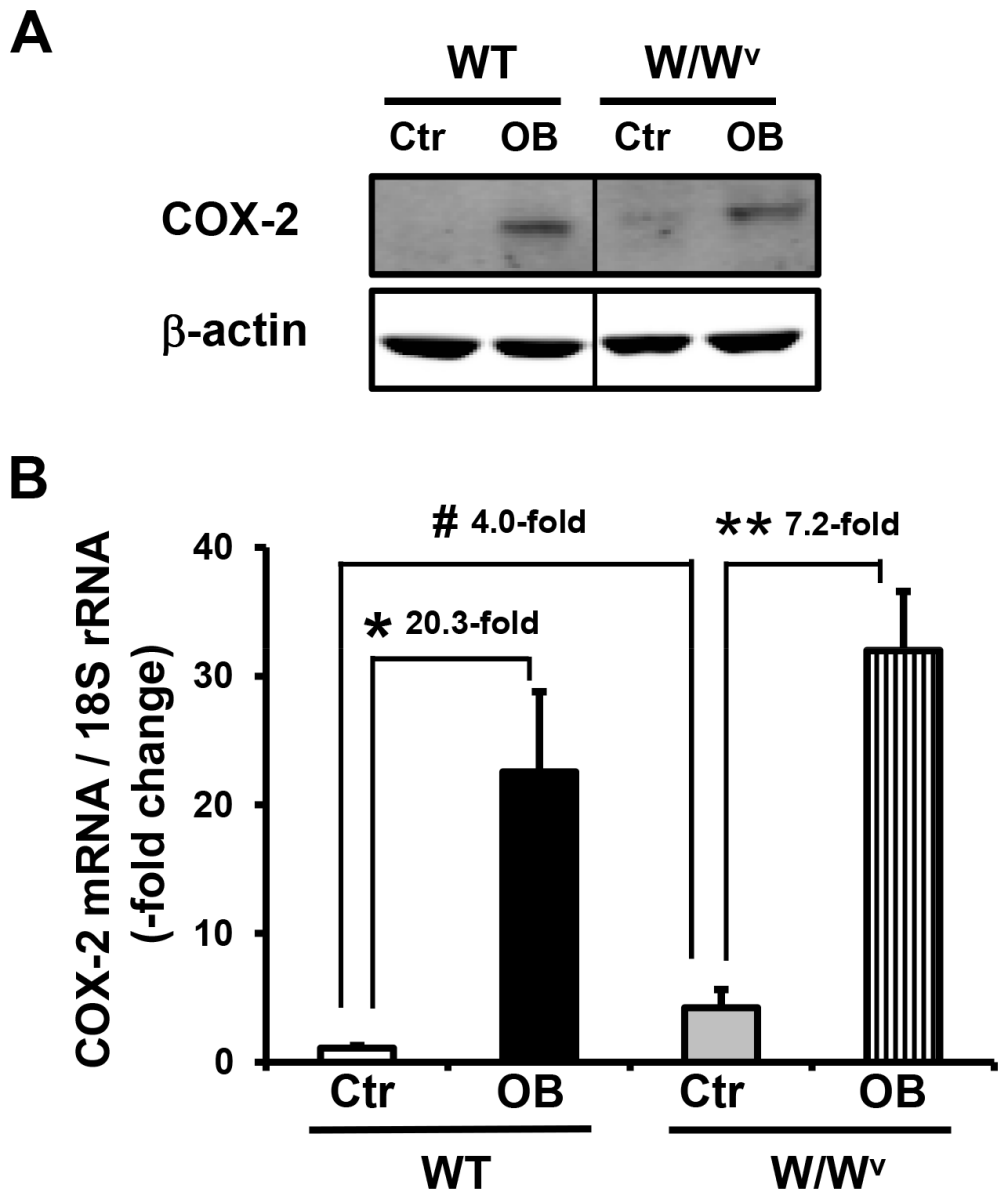
Expression of COX-2 protein (A) and mRNA (B) in wild-type and W/W^v^ mice in obstruction on day 3. Note that obstruction (OB) leads to a significant increase of COX-2 gene expression in both wild-type (WT) and W/Wv mice. Interestingly, there is significantly greater COX-2 gene expression in the W/W^v^ control mice than in wild-type control mice. N = 4 to 6. *p < 0.001 between WT Ctr vs. WT OB; **p < 0.001 between W/W^v^ Ctr vs. W/W^v^ OB; #p < 0.05 between WT Ctr and W/W^v^ Ctr. There is no significant difference between WT OB and W/W^v^ OB (p>0.05).

We found that partial colon obstruction also led to a dramatic induction of COX-2 gene expression in the ICC deficient W/W^v^ mice in the colonic muscularis externae (Figure 5). The COX-2 mRNA level increased from 4.4 + 1.3 in W/W^v^ control mice (N = 6) to 32.0 + 4.6 in W/W^v^ obstruction mice (3 day, N = 4). This represents a 7.2 + 1.0-fold change (p < 0.01). When compared to wild-type sham controls, this was a 28.8 + 4.1-fold change (p < 0.01). Interestingly, the COX-2 mRNA level in the control W/W^v^ mice was 4.0 + 1.1-fold higher than that in wild-type control mice (p < 0.05, p = 6 in each group).

Western blot detected a marked induction of COX-2 protein in obstruction in colonic muscularis externae in both wild-type and W/W^v^ mice (Figure 5).

### Obstruction decreases colonic circular muscle contractility in both wild-type and W/W^v^ mice

**Figure 6:**
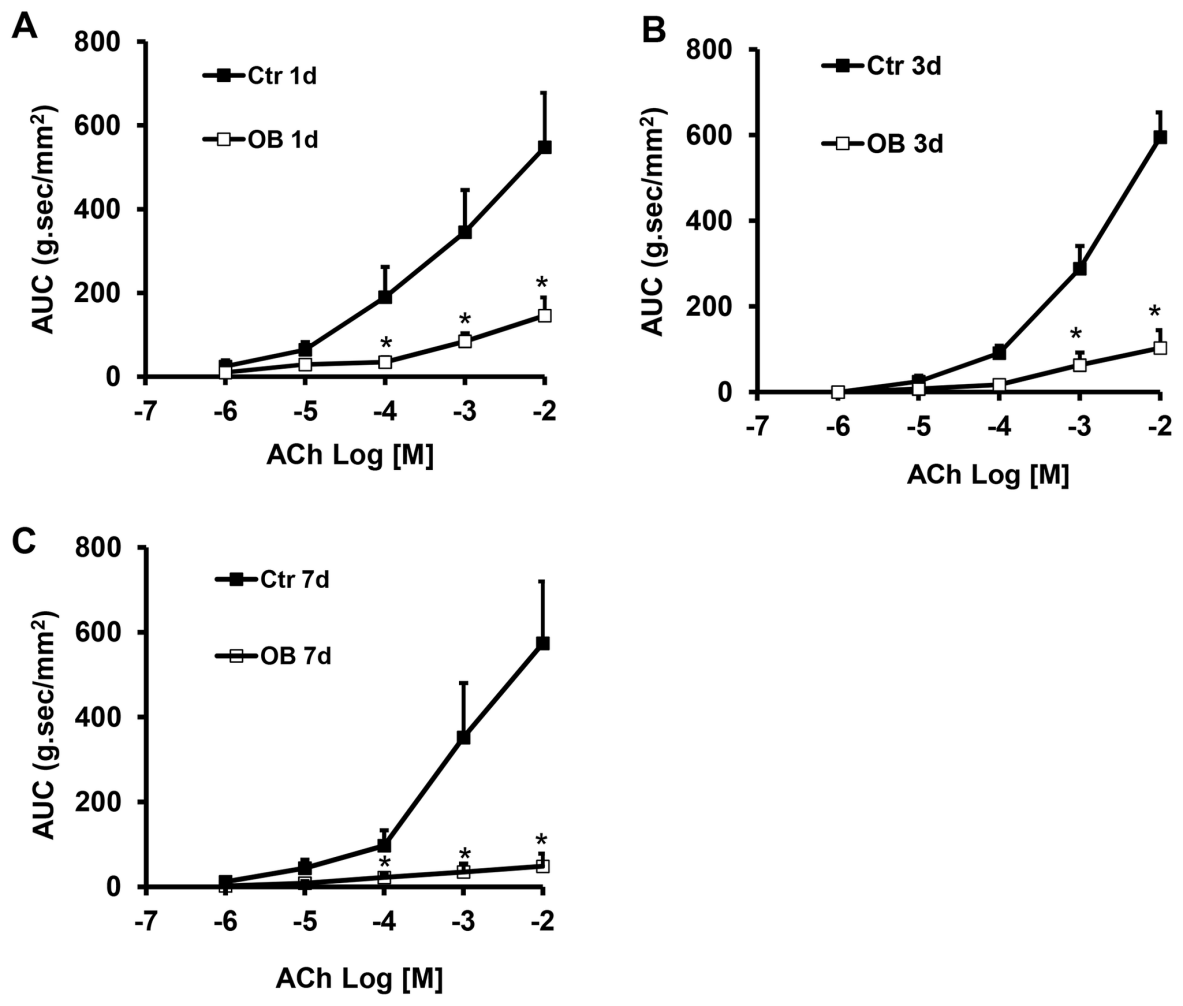
Colonic circular muscle contractility in wild-type mice in obstruction. The colonic circular muscle contractility to ACh was significantly reduced in mice with obstruction (OB) for 1 day (A, N =3), 3 days (B), and 7 days (C), compared to sham controls (Ctr). N = 3 to 5. *p < 0.05 compared to respective controls.

Muscle bath was carried out to determine colonic circular smooth muscle contractility in wild-type and W/W^v^ mice treated with sham operation and obstruction. As shown in Figure 6, the colonic circular muscle contractility to ACh was significantly impaired in obstruction in all the test time points (day1, day 3, and day 7, N = 3 or 4 animals) in the wild-type mice.

**Figure 7:**
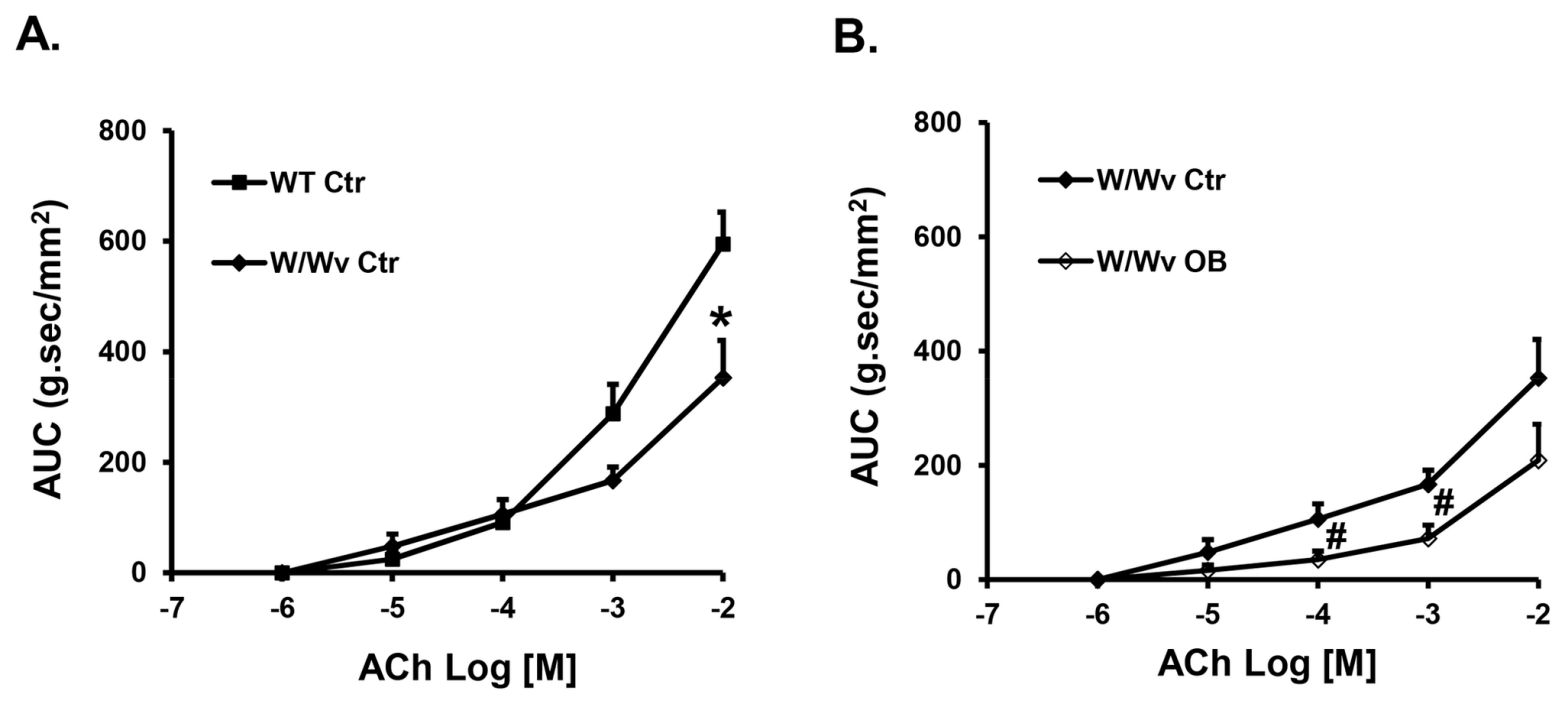
Colonic circular muscle contractility in sham operated wild-type and W/W^v^ mice (A), and the effect of obstruction (3 day) in the W/W^v^ mice (B). Note that the colonic circular muscle contractility was less in the sham W/W^v^ mice than in the wild-type sham controls. Obstruction further suppressed muscle contractility in the W/W^v^ mice. N = 3 to 5. *p < 0.05 vs. wild-type controls. P < 0.05 vs. W/W^v^ sham control.

Interestingly, the colonic circular muscle contractility was impaired in the W/W^v^ control mice compared to the wild type controls (Figure 7), especially to the higher concentrations of ACh. However, partial colon obstruction led to further suppression of colonic smooth muscle contractility in the W/W^v^ mice (Figure 7. N = 5).

### Effects of COX-2 inhibitor NS-398 in obstruction in wild-type and W/W^v^ mice

**Figure 8:**
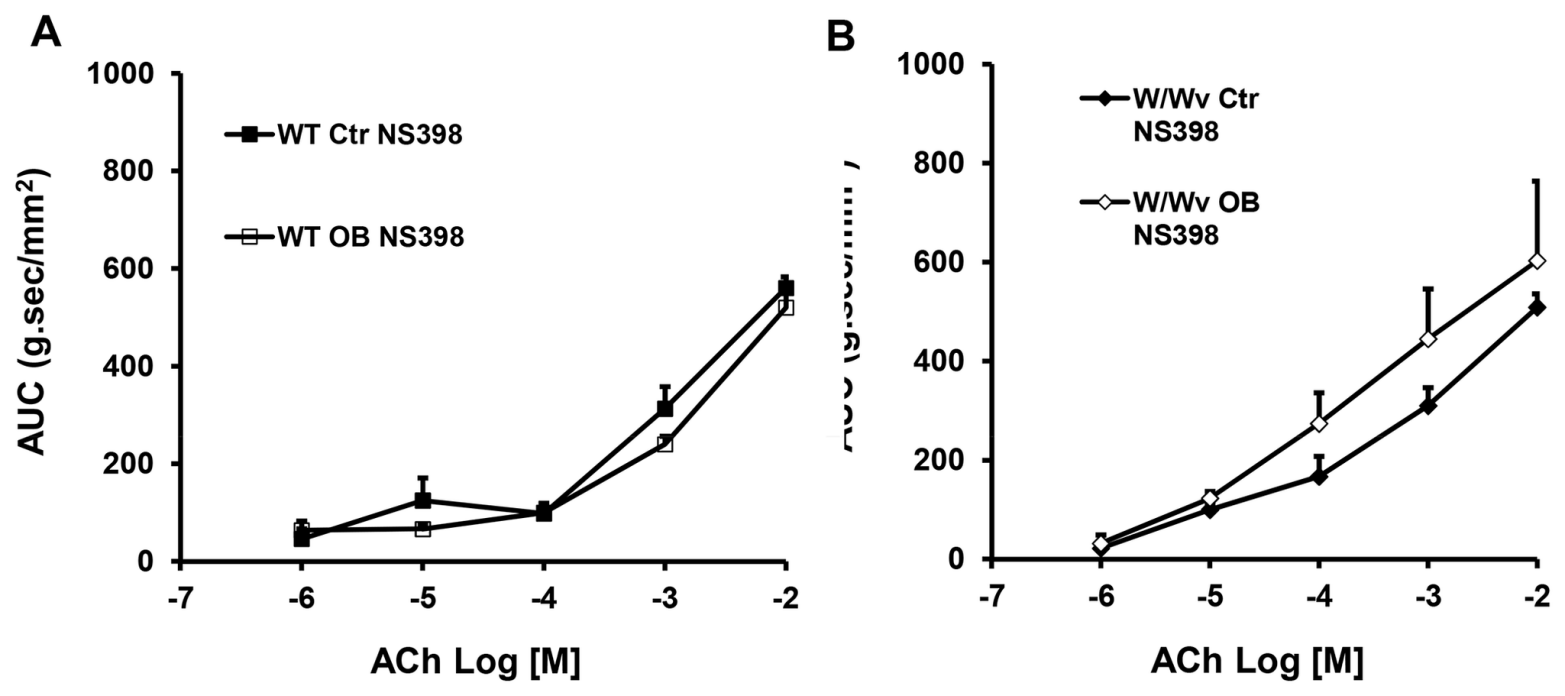
Effect of NS398 administration on colonic circular muscle contractility in obstruction (3 days) in wild-type (A) and W/W^v^ mice (B). Note that daily administration of 10 mg/kg NS398, i.p., prevented the suppression of colonic circular muscle contractility induced by obstruction in both wild-type and W/W^v^ mice. N = 4 or 5.

In another series of experiments, 10 wild-type mice (5 control and 5 obstruction) and 10 W/W^v^ mice (4 control and 6 obstruction) were given daily injections of COX-2 inhibitor NS398 (10 mg/kg, I.P.). Obstruction was maintained for 3 days. We found that in both wild-type and W/W^v^ mice, NS398 prevented the decrease of circular muscle contractility observed in obstruction (Figure 8). Interestingly, NS398 treatment increased the muscle contractility of W/W^v^ control mice to nearly the same level as in that of wild type controls (Figure 8).

## Discussion

Since interstitial cell of Cajal was postulated as a pacemaker cell in the gut in the early 1990s, many have attempted to study the possible pathophysiological roles of this cell [12]. ICC alteration was reported in many gastrointestinal conditions such as achalasia [14], gastroparesis [18], gastric outlet obstruction (hypertrophied pyloric stenosis) [15], chronic intestinal pseudo-obstruction [16], megacolon [17], and slow-transit constipation [17,22]. However, it has long been a debate whether alteration of the ICC is a cause, or a secondary effect of the disorders [12,13]. Interestingly, almost all these above mentioned conditions are associated with mechanical stress in the gut wall [5,7,8]. The loss of ICC and alteration of ICC network occurred only in the dilated segment, while the neighboring un-dilated segment showed normal distribution of ICC [23]. This was also true in animal models of bowel obstruction, as ICC was altered only in the dilated segment oral to obstruction, but not in the un-dilated distal segment [24]. Our study found that mechanical dilation led to a marked reduction of ICC density and a disruption of the ICC network in a time-dependent manner. Quantitative analysis demonstrates that the density of c-kit positive ICC was drastically decreased, and the ICC network severely disrupted by 7 days of obstruction. These data thus suggest that alteration of ICC in obstructive conditions is caused by mechanical stress, and a loss of ICC is less likely the primary pathogenic cause for OBD. This may also speak for the fact that ICC alteration has been reported non-discriminatingly in almost all types of OBD.

As shown in the results and in the previous studies [5], obstruction led to marked lumen dilation in the proximal segment starting a few hours after the introduction of obstruction band. One may argue that the quantitative metric value changes of ICC network (decreased density, increased hole size, and damaged connectivity) may be simply due to a passive effect of mechanical stretch, rather than a patho-physiological alteration. However, the destructive changes of ICC network did not occur on day 1 or day 3 when lumen distension was apparently present. Furthermore, the thickness of the ICC network was not significantly changed in all the tested time points (up to 7 days). These data indicate that these time-dependent changes of ICC network are not a passive effect of mechanical stretch, but a true patho-physiological alteration induced by lumen distention.

Motility dysfunction is well documented in patients with OBD [2-4]. In animal studies in rats and mice [5,7,25], gut smooth muscle contractility was significantly suppressed in obstruction. Our previous studies demonstrated that mechanical stress in obstruction induced gene expression of COX-2, and this mechano-transcription process plays a critical role in the impairment of smooth muscle contractility [5,6,8]. We found that stretch-induced expression of COX-2 in bowel obstruction is a smooth muscle specific phenomenon. Immunohistochemical studies [5] showed that up-regulation of COX-2 expression occurs in the smooth muscle cells only, not in the mucosa layer, or submucosa, or myenteric plexus where the ICCs are located. These data suggest that smooth muscle cells are highly mechanosensitive in conditions such as bowel obstruction, and serve as a critical player in mechano-transcription in the gut. As some earlier studies suggested that ICC might be mechanoreceptors in the gut [11], we determined if c-kit positive ICC are required in the process of mechano-transcription in the colon by comparing the expression of COX-2 gene in obstruction in wild-type and in ICC deficient mice. Our data suggest that ICC are not required in obstruction-induced mechanotranscription of COX-2 in the colon. As in the wild-type mice, lumen dilation in bowel obstruction markedly induced expression of COX-2 mRNA in W/W^v^ mice. Compared to the wild-type sham control mice, obstruction for 3 days led to a 20-fold increase of COX-2 mRNA in the colonic muscularis externae in the wild-type mice, and a 29-fold increase in the ICC deficient W/W^v^ mice.

Our study also offers several lines of evidence suggesting that c-kit positive ICC may not be involved in obstruction-induced impairment of smooth muscle contractility. First of all, impaired smooth muscle contractility was observed in obstruction in the wild-type mice as early as 1 day after the introduction of an obstruction band, when the ICC network was histologically normal. Secondly, obstruction led to suppression of smooth muscle contractility also in the ICC deficient mice. Furthermore, we found that induction of COX-2 gene expression is mainly accounted for the suppression of smooth muscle contractility in obstruction. Expression of COX-2 gene was up-regulated in obstruction in wild-type mice, and in the W/Wv mice. Inhibition of COX-2 activity with NS-398 significantly improved smooth muscle contractile function in obstruction in both the wild-type and ICC deficient mice.

It is noteworthy that the COX-2 gene expression level is significantly higher and muscle contractility is less responsive in the colonic muscularis in W/Wv control mice compared to wild-type control mice. Inhibition of COX-2 activity with NS398 normalized the muscle contractility of W/W^v^ control mice, suggesting an important role of COX-2, rather than deficiency of ICC, in the impairment of colonic contractility in W/W^v^ mice. The reason for the increased COX-2 expression in W/W^v^ mice is not clear at the time. However, an increased expression of COX-2 in the gastric tissue has also been reported in the W/W^v^ mice (Winston, Chen, Shi, Sarna. unpublished observation). As a matter of fact, other proinflammatory molecules such as iNOS are also increased in the gastric tissue in W/W^v^ mice. This may suggest that a general proinflammatory response is present in the gastrointestinal tract in the c-kit gene deficient animals.

In summary, our study evaluated the role of ICC in obstructed colon in mice. We demonstrated that the ICC network is altered as a result of obstruction in a time-dependent manner. Furthermore, the c-kit positive ICC are not required in mechanical stress-induced gene expression of COX-2, and do not appear to be involved in the impairment of smooth muscle contractility in partial colon obstruction in mice. Instead, our data suggest that stretch-induced COX-2 is the primary culprit for the impaired contractility in obstruction.

## References

[1] Welch JP (1990) General considerations and mortality. In: Welch JP Bowel obstruction. Saunders, Philadelphia, PA. . pp. 59-95.

[2] Jordan GL Jr (1980) The acute abdomen. Adv Surg 14: 259-315. PubMed: 7008548. 7008548

[3] Russell JC, Welch JP (1990) Pathophysiology of Bowel Obstruction. In: Welch JP Bowel Obstruction. Saunders, Philadelphia, PA. pp. 28-58.

[4] Summers RW (1999) Chapter 39, Approach to the patient with ileus and obstruction. In: Yamada T Alpers DH Laine L Owyang C Powell DW Textbook of Gastroenterology, Vol.1. Lippincott Williams & Wilkins, Philadelphia, PA. pp.715-731.

[5] Shi XZ, Lin YM, Powell DW, Sarna SK (2011) Pathophysiology of motility dysfunction in bowel obstruction: role of stretch-induced COX-2. Am J Physiol Gastrointest Liver Physiol 300: G99–108. doi:10.1152/ajpgi.00379.2010. PubMed: 21051526. 21051526 10.1152/ajpgi.00379.2010 PMC3025501

[6] Lin YM, Sarna SK, Shi XZ (2012) Prophylactic and therapeutic benefits of COX-2 inhibitor on motility dysfunction in bowel obstruction: roles of PGE_₂_ and EP receptors. Am J Physiol Gastrointest Liver Physiol 302(2): G267-G275. doi:10.1152/ajpgi.00326.2011. PubMed: 22038825. 22038825 10.1152/ajpgi.00326.2011 PMC3341114

[7] Lin YM, Li F, Shi XZ (2012) Mechanotranscription of COX-2 is a common response to lumen dilation of the rat gastrointestinal tract. Neurogastroenterol Motil 24(7): 670-677, e295-6 doi:10.1111/j.1365-2982.2012.01918.x. PubMed: 22489918. 22489918 10.1111/j.1365-2982.2012.01918.x PMC4183192

[8] Li F, Lin YM, Sarna SK, Shi XZ (2012) Cellular mechanism of mechanotranscription in colonic smooth muscle cells. Am J Physiol Gastrointest Liver Physiol 303(5): G646-G656. doi:10.1152/ajpgi.00440.2011. PubMed: 22700825. 22700825 10.1152/ajpgi.00440.2011 PMC3468553

[9] Sanders KM, Ordög T, Koh SD, Torihashi S, Ward SM (1999) Development and plasticity of interstitial cells of Cajal. Neurogastroenterol Motil 11: 311-338. doi:10.1046/j.1365-2982.1999.00164.x. PubMed: 10520164. 10520164 10.1046/j.1365-2982.1999.00164.x

[10] Thomsen L, Robinson TL, Lee JCF, Farraway LA, Hughes MJG et al. (1998) Interstitial cells of Cajal generate a rhythmic pacemaker current. Nat Med 4: 848-851. doi:10.1038/nm0798-848. PubMed: 9662380. 9662380 10.1038/nm0798-848

[11] Won KJ, Sanders KM, Ward SM (2005) Interstitial cells of Cajal mediate mechanosensitive responses in the stomach. Proc Natl Acad Sci U S A 102: 14913-14918. doi:10.1073/pnas.0503628102. PubMed: 16204383. 16204383 10.1073/pnas.0503628102 PMC1253552

[12] Sarna SK (2008) Are interstitial cells of Cajal plurifunction cells in the gut? Am J Physiol Gastrointest Liver Physiol 294: G372-G390. PubMed: 17932226. 17932226 10.1152/ajpgi.00344.2007

[13] Goyal RK, Chaudhury A (2010) Mounting evidence against the role of ICC in neurotransmission to smooth muscle in the gut. Am J Physiol Gastrointest Liver Physiol 298: G10-G13. doi:10.1152/ajpgi.00426.2009. PubMed: 19892937. 19892937 10.1152/ajpgi.00426.2009 PMC2806097

[14] Faussone-Pellegrini MS, Cortesini C (1985) The muscle coat of the lower esophageal sphincter in patients with achalasia and hypertensive sphincter. An electron microscopic study. J Submicrosc Cytol 17: 673-685. PubMed: 4078952. 4078952

[15] Isozaki K, Hirota S, Miyagawa J, Taniguchi M, Shinomura Y et al. (1997) Deficiency of c-kit+ cells in patients with a myopathic form of chronic idiopathic intestinal pseudo-obstruction. Am J Gastroenterol 92: 332-334. PubMed: 9040218. 9040218

[16] Vanderwinden JM, Liu H, De Laet MH, Vanderhaeghen JJ (1996) Study of the interstitial cells of Cajal in infantile hypertrophic pyloric stenosis. Gastroenterology 111: 279-288. doi:10.1053/gast.1996.v111.pm 8690192. PubMed: 8690192 8690192 10.1053/gast.1996.v111.pm8690192

[17] Wedel T, Spiegler J, Soellner S, Roblick UJ, Schiedeck THK et al. (2002) Enteric Nerves and Interstitial Cells of Cajal Are Altered in Patients with Slow-Transit Constipation and Megacolon. Gastroenterology 123: 1459-1467. doi:10.1053/gast.2002.36600. PubMed: 12404220. 12404220 10.1053/gast.2002.36600

[18] Zárate N, Mearin F, Wang XY, Hewlett B, Huizinga JD et al. (2003) Severe idiopathic gastroparesis due to neuronal and interstitial cells of Cajal degeneration: pathological findings and management. Gut 52: 966-970. doi:10.1136/gut.52.7.966. PubMed: 12801952. 12801952 10.1136/gut.52.7.966 PMC1773724

[19] Gao J, Du P, Archer R, O’Grady G, Archer R et al. (2013) Numerical metrics for automated quantification of interstitial cell of Cajal network structural properties. J R Soc Interface 10: . (2013) PubMed: 23804441. 10.1098/rsif.2013.0421 PMC3730694 23804441

[20] Shi XZ, Choudhury BK, Pasricha PJ, Sarna SK (2007) A novel role of VIP in colonic motility function: induction of excitation-transcription coupling in smooth muscle cells. Gastroenterology 132(4): 1388-1400. doi:10.1053/j.gastro.2007.02.016. PubMed: 17408637. 17408637 10.1053/j.gastro.2007.02.016

[21] Okishio Y, Takeuchi T, Fujita A, Suenaga K, Fujinami K et al. (2005) Ascending contraction and descending relaxation in the distal colon of mice lacking interstitial cells of Cajal. J Smooth Muscle Res 41(3): 163-174. doi:10.1540/jsmr.41.163. PubMed: 16006749. 16006749 10.1540/jsmr.41.163

[22] He CL, Burgart L, Wang L, Pemberton J, Young-Fadok T, Szurszewski J, Farrugia G (2000) Decreased interstitial cell of cajal volume in patients with slow-transit constipation. Gastroenterology 118(1): 14-21. doi:10.1016/S0016-5085(00)70409-4. PubMed: 10611149. 10611149 10.1016/s0016-5085(00)70409-4

[23] Okada T, Sasaki F, Honda S, Cho K, Matsuno Y et al. (2010) Disorders of interstitial cells of Cajal in a neonate with segmental dilatation of the intestine. J Pediatr Surg 45(6): e11-e14. doi:10.1016/j.jpedsurg.2010.03.024. PubMed: 20620293. 10.1016/j.jpedsurg.2010.03.024 20620293

[24] Chang IY, Glasgow NJ, Takayama I, Horiguchi K, Sanders KM et al. (2001) Loss of interstitial cells of Cajal and development of electrical dysfunction in murine small bowel obstruction. J Physiol 5362: 555-568. doi:10.1111/j.1469-7793.2001.0555c.xd. PubMed: 11600689. 11600689 10.1111/j.1469-7793.2001.0555c.xd PMC2278884

[25] Won KJ, Suzuki T, Hori M, Ozaki H (2006) Motility disorder in experimentally obstructed intestine: relationship between muscularis inflammation and disruption of the ICC network. Neurogastroenterol Motil 18(1): 53-61. doi:10.1111/j.1365-2982.2005.00718.x. PubMed: 16371083. 16371083 10.1111/j.1365-2982.2005.00718.x

